# Sex differences in stress-induced social withdrawal: role of brain derived neurotrophic factor in the bed nucleus of the stria terminalis

**DOI:** 10.3389/fnbeh.2013.00223

**Published:** 2014-01-09

**Authors:** Gian D. Greenberg, Abigail Laman-Maharg, Katharine L. Campi, Heather Voigt, Veronica N. Orr, Leslie Schaal, Brian C. Trainor

**Affiliations:** ^1^Neuroscience Graduate Group, University of CaliforniaDavis, CA, USA; ^2^Department of Psychology, University of CaliforniaDavis, CA, USA; ^3^Center for Neuroscience, University of CaliforniaDavis, CA, USA

**Keywords:** BDNF, BNST, social defeat, SSRI, sex, dose

## Abstract

Depression and anxiety disorders are more common in women than men, and little is known about the neurobiological mechanisms that contribute to this disparity. Recent data suggest that stress-induced changes in neurotrophins have opposing effects on behavior by acting in different brain networks. Social defeat has been an important approach for understanding neurotrophin action, but low female aggression levels in rats and mice have limited the application of these methods primarily to males. We examined the effects of social defeat in monogamous California mice (*Peromyscus californicus*), a species in which both males and females defend territories. We demonstrate that defeat stress increases mature brain-derived neurotrophic factor (BDNF) protein but not mRNA in the bed nucleus of the stria terminalis (BNST) in females but not males. Changes in BDNF protein were limited to anterior subregions of the BNST, and there were no changes in the adjacent nucleus accumbens (NAc). The effects of defeat on social withdrawal behavior and BDNF were reversed by chronic, low doses of the antidepressant sertraline. However, higher doses of sertraline restored social withdrawal and elevated BDNF levels. Acute treatment with a low dose of sertraline failed to reverse the effects of defeat. Infusions of the selective tyrosine-related kinase B receptor (TrkB) antagonist ANA-12 into the anterior BNST specifically increased social interaction in stressed females but had no effect on behavior in females naïve to defeat. These results suggest that stress-induced increases in BDNF in the anterior BNST contribute to the exaggerated social withdrawal phenotype observed in females.

## Introduction

Mood disorders such as depression are diagnosed more frequently in women than men (Kessler et al., 1994; Kessler, 2003). Commonly prescribed antidepressants also differ in efficacy (Fava and Rankin, 2002), with women showing greater sensitivity to selective serotonin reuptake inhibitors (SSRIs) (Khan et al., 2005; Young et al., 2009). However, the mechanisms underlying these sex differences are largely unknown, as the majority of preclinical studies focus exclusively on males (Cryan and Mombereau, 2004; LaPlant et al., 2009). The social aversion phenotype induced by social defeat stress (Krishnan et al., 2007; Tamashiro et al., 2007; Anstrom et al., 2009; Miczek et al., 2011; Trainor et al., 2011) has become a widely used marker for SSRI efficacy, as it is reversed by chronic but not acute antidepressant treatment (Berton et al., 2006; Tsankova et al., 2006; Cao et al., 2010; Venzala et al., 2012; Warren et al., 2012). However, sex differences in defeat induced social aversion have been understudied, as female aggression levels are low in most laboratory rodent species (but see: Solomon et al., 2007; Holly et al., 2012). Female California mice (*Peromyscus californicus*) aggressively defend home territories in lab settings (Silva et al., 2010). In this species, three episodes of social defeat induce social aversion in females but not males (Trainor et al., 2011, 2013). Previous work in male rodents has suggested that neurotrophins, including brain-derived neurotrophic factor (BDNF), are important mediators of social aversion (Russo and Nestler, 2013).

Social aversion is associated with decreased BDNF protein in hippocampus (Tsankova et al., 2006), and chronic fluoxetine treatment reverses this effect (Hodes et al., 2010). In contrast, defeat *increases* BDNF protein in the nucleus accumbens (NAc), and chronic SSRI treatment reverses this upregulation of BDNF (Berton et al., 2006; Krishnan et al., 2007, 2008). Thus, the connection between neurotrophins and depression-like behaviors is nuanced (Berton et al., 2006; Taylor et al., 2011). The NAc and hippocampus lack dramatic anatomical sex differences. However, posterior subregions of the bed nucleus of the stria terminalis (BNST) have striking neuroanatomical (Del Abril et al., 1987; Chung et al., 2000; McCarthy, 2009; Murray et al., 2009) and neurochemical (Han and de Vries, 2003; Shah et al., 2004; Wu et al., 2009) sex differences. In contrast, anterior subregions do not display neuroanatomial size differences (Campi et al., 2013), but they do have important effects on long-term behavioral responses to stress (Aguilera et al., 1987; Han and de Vries, 2003; Bangasser and Shors, 2008). Intriguingly, recent evidence suggests female rats, but not males, have increased neuronal activity in the anterior BNST following restraint stress (Babb et al., 2013). Both anterior and posterior subregions have important effects on social behavior (Newman, 1999).

We demonstrate that social defeat stress increases mature BDNF protein but not mRNA in the BNST of female California mice but not males. This effect was localized to the anterior but not posterior BNST and is consistent with previous studies showing that activity in BNST is necessary for defeat-induced changes in social behavior (Jasnow et al., 2004; Markham et al., 2009). Chronic but not acute treatment with a low dose of sertraline reversed the social withdrawal phenotype in females exposed to defeat and normalized BDNF levels. Finally, blocking BDNF signaling in the anterior BNST with infusions of a selective tyrosine-related kinase B (TrkB) antagonist increased social interaction behavior in stressed females. These findings have important implications for neurotrophin hypotheses of mental disorders.

## Materials and methods

### Animals

Male and female California mice (*Peromyscus californicus*) used for these experiments were bred in our laboratory colony. In genome mapping studies, other species within the *Peromyscus* genus were more similar to laboratory rats (*Rattus norvegicus*) than mice (*Mus musculus)* (Ramsdell et al., 2008). Unlike most species of mammals, California mice are monogamous and biparental (Ribble, 1991; Gubernick and Teferi, 2000). Female California mice defend nest sites in natural environments (Ribble and Salvioni, 1990), and they are aggressive toward other females in laboratory settings (Davis and Marler, 2003; Silva et al., 2010). These high levels of aggression allow for the use of social defeat methods in both male and female California mice. Three episodes of social defeat induce social aversion in females (Trainor et al., 2013).

California mice are nocturnal, so behavioral testing for all experiments was conducted during the dark phase under dim red light. Estrous cycles were not monitored continuously because social aversion is observed across all stages of the estrous cycle (Trainor et al., 2011), and ovariectomy has no effect on defeat-induced social withdrawal behavior (Trainor et al., 2013). Animals were housed in same sex groups of 2–3 per cage on 300 ml corncob bedding (1/8 in.; Andersons, Maume, OH; no. 88) with cotton nestlets in clear polypropylene cages, and they were kept on a 16 h light/8 h dark cycle (lights on 2300 h) with free access to Harlan Teklad 2016 food (Hayward, CA) and water. All procedures in this study were consistent with the NIH Guide for the Care and Use of Laboratory Animals and approved by the Institutional Animal Care and Use Committee (IACUC) at the University of California, Davis.

### Behavioral testing

#### Social defeat stress

Adult mice (>3 months old) were randomly assigned to social defeat or control handling for 3 consecutive days (Trainor et al., 2011, 2013). Mice assigned to social defeat were introduced to the home cage of an aggressive, same-sex sexually-experienced mouse during the dark phase. Episodes of defeat were terminated following either 7 min or 10 bites from the resident, whichever occurred first. Control mice were introduced to an empty cage for 7 min. This approach more closely resembles methods used in rats (Carnevali et al., 2012; Holly et al., 2012; Nikulina et al., 2012) and Syrian hamsters (*Mesocricetus auratus)* (Taylor et al., 2011; Morrison et al., 2012). In these species, significant changes in brain and behavior are observed after only 1–5 social defeat episodes. This contrasts with widely used social defeat methods in *M. musculus*, which combine 10 episodes of defeat with sensory contact with an aggressor (Berton et al., 2006; Iñiguez et al., 2010; Warren et al., 2012). Male *M. musculus* appear to be somewhat resistant to defeat, as mice exposed to three episodes of defeat on 1 day do not exhibit social withdrawal (Krishnan et al., 2007; Christoffel et al., 2011).

#### Open field and social interaction testing

Social interaction tests consisted of three phases, 3 min each (Trainor et al., 2013). In the open field phase (OFT), animals were allowed to explore a large open field (Figure 2A, 89 × 63 × 60 cm). This relatively large arena accommodates the larger size of California mice (~35–40 g). Time spent within 8 cm of the sides and within a center zone located 14 cm from the sides were recorded using the Any-Maze video tracking system (Stoelting, Wood Dale, IL). During the acclimation phase a small wire cage was introduced against one side of the arena, and the amount of time the mouse spent within 8 cm of the empty cage was recorded. During the social interaction phase an unfamiliar, same-sex virgin stimulus mouse was placed into the wire cage. We recorded the amount of time the focal mouse spent interacting with the wire cage and the duration spent in the two corners opposite the wire cage. Total distance traveled during the OFT was measured as an estimate of total activity. Social interaction testing was conducted 2–4 weeks after defeat or handling because we previously observed that effects of defeat are stronger at these time points compared to 1 day after defeat (Trainor et al., 2011).

### Experiment 1: effects of defeat on brain and behavior

**Figure 1:**
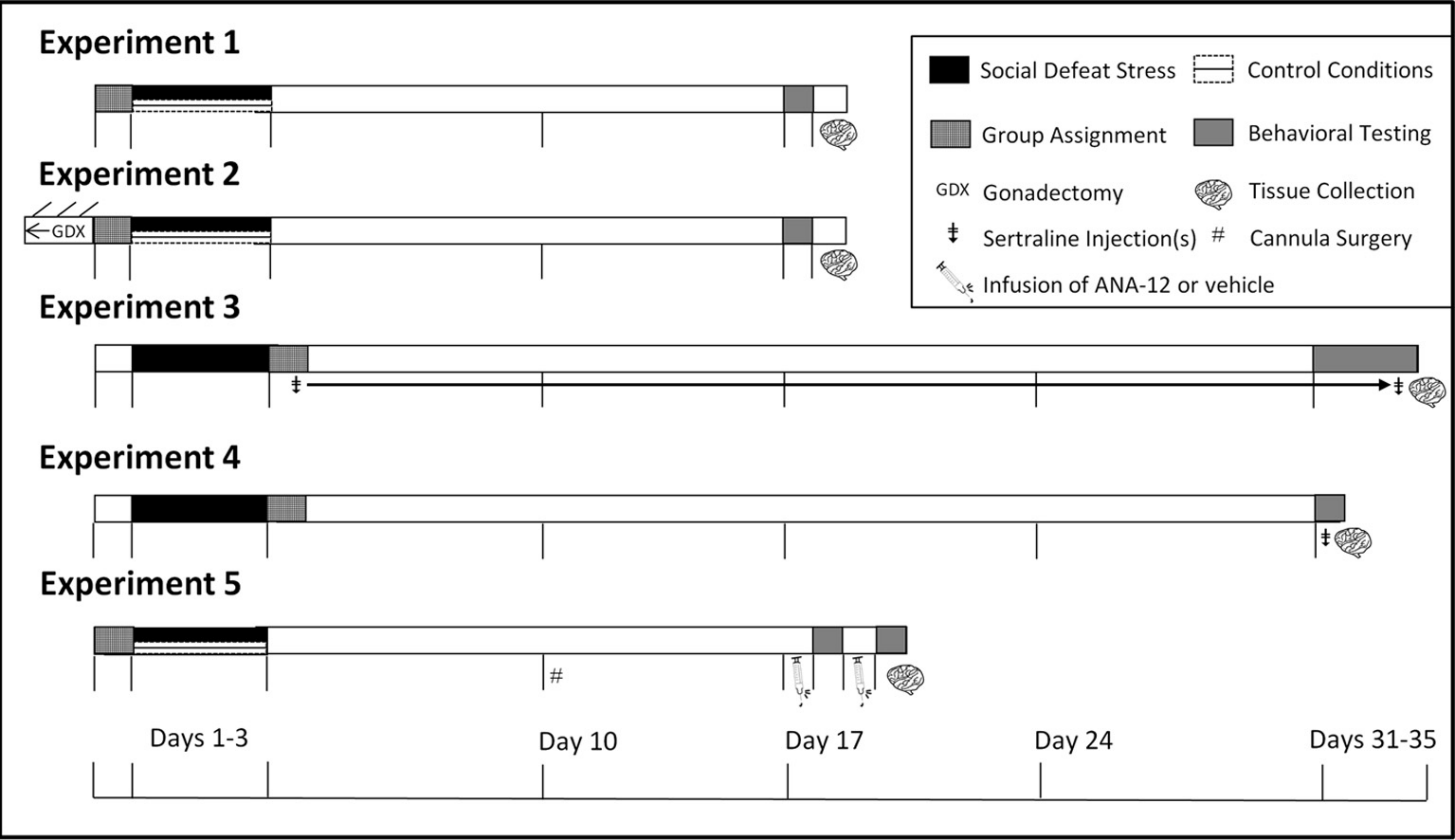
**Experimental design and timeline**. In Experiments 1 and 2 mice were tested in social interaction tests (day 17). In Experiment 3 mice were tested in social interaction tests (day 31), sucrose anhedonia (days 32–34), and forced swim (day 35). In Experiment 4, mice were tested in forced swim 1 h following social interaction (day 31). In Experiment 5 mice were tested in social interaction twice due to within-subjects design and were euthanized immediately following the second day of social interaction testing. ^‡^Indicates injection(s) of sertraline. ^GDX^Indicates gonadectomy. ^#^Indicates cannula surgeries.

Three sets of mice underwent social defeat or control conditions. Between the final defeat episode and the social interaction test (days 4–17, Figure 1), mice were undisturbed except for weekly cage changes and weight monitoring. In the first set, mice were tested in social interaction tests on day 17 (2-weeks post-final defeat episode). On the morning following social interaction testing (day 18), these mice were anesthetized with isoflurane and euthanized by decapitation (1100 h). Brains were removed and 2 mm-thick coronal sections were made using a brain matrix. The BNST was dissected using 1 mm-diameter bilateral punches and then frozen on dry ice (Greenberg et al., 2012). These samples were used to quantify BDNF protein across the entire anterior-posterior extent of the BNST. A second set of mice was assigned to control or defeat conditions and tested in social interaction tests on day 17. These mice were euthanized by isoflurane anesthesia and decapitation. Brains were frozen on dry ice and then sectioned at 500 μm on a cryostat. Sections were stored overnight in RNAlater (AMBION, Austin, TX) at 4°C before 1 mm-diameter bilateral punches were taken of the anterior BNST, dorsal-posterior BNST and NAc. These punch samples were used to quantify BDNF protein with Western blot. A third set of mice was used for BDNF immunohistochemistry and were tested the same way as mice in the previous two sets. After social interaction testing these mice were anesthetized with Beuthanasia-D (250 mg/kg, i.p., Schering, Union, NJ) and rapidly perfused with ice cold 4% paraformaldehyde solution. Brains were extracted and submerged in 20% sucrose in 0.1 M PBS overnight. They were then frozen at −40°C.

#### Western blotting

Punch samples were homogenized in ice cold buffer (7.4 pH, 20% glycerol, 0.4 M NaCl, 20 mM HEPES, 5 mM MgCl_2_, 0.5 mM EDTA in H_2_O) with protease inhibitor (1% PMSF in EtOH). Laemmli buffer (Sigma, St. Louis, MO) was added to the homogenate at a 1:1 dilution and samples were placed on a shaker at 4°C for 1 h. Proteins were denatured at 98°C for 5 min, chilled on ice, and separated with gel electrophoresis (15% bis-acrylamide resolving gel). Protein was transferred to polyvinylidine fluoride (PVDF) membranes (Bio-Rad, Hercules, CA), rinsed, and blocked with 5% skim milk in 0.1%Triton-X with tris-buffered saline (TBS-T). Membranes were incubated overnight in primary rabbit anti-BDNF (sc-546, Santa Cruz Antibodies, 1:900) at 4°C. Antibody specificity was determined by running a recombinant BDNF peptide (Genway, San Diego, CA, Figure 3A). Membranes were then incubated for 1 h in peroxidase-conjugated anti-rabbit secondary antibody (Vector, Burlingame, CA) diluted 1:2000 in TBS-T. Following TBS-T washes, developing solution was applied (Bio-Rad), and the blot was imaged on a Bio-Rad ChemiDoc. We quantified the mature form of the BDNF protein at ~14 kDa (Figure 3A). Blots were probed for β-actin as a loading control (Cell Signaling, Danvers, MA, 1:2000), and BDNF protein bands were normalized to their respective actin controls. BDNF/actin ratios were further normalized to control mice on each blot. Levels of protein are displayed as a percentage of the levels quantified from control males on each blot.

#### Immunohistochemistry

Brains were sectioned on a cryostat at 40 μm and stored in cryoprotectant (50% v/v phosphate buffer, 30% w/v sucrose, 1% w/v polyvinylprolidone, 30% v/v ethylene glycol) at −20°C. Every third section was processed for immunohistochemistry. Following antigen retrieval with pepsin treatment (0.15 mg/ml in 0.1 N HCL) for 1 min at 37°C, sections were washed three times in PBS. Endogenous peroxidases were quenched by submerging sections in H_2_O_2_ (0.5% in PBS) for 30 min. Sections were then washed three times with PBS and blocked in 3% normal goat serum (NGS) in PBS with triton × (PBS-TX, 0.2%) on an orbital shaker overnight at 4°C. On day 2, tissue was washed three times in PBS and then incubated in rabbit anti-brain-derived neurotrophic factor (BDNF) antibody (sc-546, Santa Cruz, 1:400) diluted with 2% NGS in PBS-TX (0.5%) for 24 h. Sections were washed again 3 times in PBS and incubated in biotinylated goat-anti-rabbit antibody in 2% NGS in PBS with 0.5% TX (Vector Laboratories, 1:1000) for 2 h. Sections were washed three times in PBS and incubated in avidin-biotin complex (ABC Elite Kit, Vector Laboratories) for 30 min. Sections were then washed three times in PBS and developed in nickel enhanced diaminobenzidine (Vector Laboratories) for 2 min. Sections were then rinsed in PBS and mounted onto plus slides (Fisher, Pittsburgh, PA). Slides were dehydrated in ethanol followed by Histoclear (National Diagnostics, Atlanta, GA) and coverslipped with Permount (Fisher).

We captured images with a Zeiss Axioimager. Image backgrounds were normalized by adjusting the exposure time. Both the left and right side of 2 sections containing the NAc were quantified for BDNF immunoreactivity (Figure 4A). Percent staining of BDNF was quantified in a frame of uniform size (NAc core, 0.3 × 0.29 mm; NAc shell, 0.3 × 29 mm) using Image J (NIH, Bethesda, MD) by an observer unaware of treatment assignments. Percent staining was determined using the threshold function of Image J. Six sections spanning the entire anterior BNST were quantified. Analysis was further subdivided into three pairs of sections, corresponding to the anterior, middle, and posterior portions of the anterior BNST (Figure 4A). The left and right side of each section were quantified. We used a uniform box size (0.6 × 0.6 mm) to calculate percent staining in anterior medial (BNST_AM_), anterior lateral (BNST_AL_), and anterior ventromedial BNST (BNST_VM_).

### Experiment 2—effects of defeat on *Bdnf* mRNA in the BNST

In this study, we analyzed punch samples of the BNST from a previous study examining the effects of gonadectomy on defeat-induced social withdrawal (Trainor et al., 2013). Male and female California mice were randomly assigned to gonadectomy or sham surgery (Figure 1). After 4 weeks of recovery, mice were again randomly assigned to defeat or control handling as described in Experiment 1. Two weeks later, mice were tested in social interaction tests and then immediately euthanized. Punch samples (1 mm-diamter) from 2 mm-thick sections were collected from the BNST and frozen on dry ice. RNA was extracted with RNaqueous kits (Life Tech) and then reverse transcribed. Specific primers and Taqman probe were designed based on a sequence of the California mouse *Bdnf* mRNA (Genbank Accession: JX977026, Table 1). We then used an ABI 7500 Sequencing Detection system (Applied Biosystems, Foster City, CA) and Taqman chemistry to detect *Bdnf* mRNA as well as an 18 s ribosomal RNA assay (Life Tech). Relative gene expression was calculated by comparison to standard curves consisting of serial dilutions of pooled California mouse brain cDNA followed by normalization to 18 s gene expression. Gene expression was normalized to a cDNA pool run on each plate.

**Table 1:** **Primer and probe combinations for BDNF real-time PCR**.

**Accession #**	**F primer**	**R primer**	**Probe**
JX977026	CCATAAGGACGCGGACTTGTAT	GCAGAGGAGGCTCCAAAGG	CTTCCCGGGTGATGCTCAGCAGTC

### Experiment 3: sex differences in dose-response of chronic antidepressant treatment following social defeat stress

#### Drugs and behavioral testing

Sertraline hydrochloride (Matrix Scientific, Columbia, South Carolina) was dissolved in vehicle consisting of filtered phosphate-buffered saline (PBS) with 10% Tween 80 (polysorbate 80). Injections were prepared fresh daily in 0.1 mL vehicle and administered subcutaneously (s.c.) at one of four doses of sertraline (0, 5, 10, or 20 mg/kg) for 4 consecutive weeks (days 4–35, Figure 1) between 1000 and 1200 h daily. Doses are based on previous mouse (Berton et al., 2006; Jacobsen et al., 2008) and rat (Detke et al., 1995) studies. Males and females were exposed to 3 days of social defeat stress (days 1–3). During day 31 of the study (Figure 1), the social interaction test was conducted as described in Experiment 1 to assess social investigation.

***Sucrose anhedonia test.*** During days 32–34 of the study, mice were tested for sucrose preference. Mice were pre-exposed to two water bottles filled with 1% (w/v) sucrose solution for 3 days (days 29–31) to habituate them to drinking sucrose and to drinking from two sides of a cage (Green et al., 2006; Wallace et al., 2009). During each preference test day (days 32–34), mice were individually housed from “lights out” (1500) to “lights on” (2300). Two pre-weighted water bottles, filled with sucrose (1%, dissolved in tap water) or tap water only were introduced 30 min before lights out, and fluid intake was recorded from each bottle 8 h later. Immediately following each sucrose testing session (2300), mice were returned to group housing with their original cage mates. Percent sucrose consumption was measured by dividing the mass of sucrose solution consumed by the total mass of liquid consumed (water + sucrose). The side placement of water and sucrose bottles was switched every other day to reduce confounds from side biases.

***Forced swim and euthanasia.*** During day 35 of the study, mice were tested in the forced swim test. Mice were placed in a cylindrical tank (24 cm diameter, 53 cm height) filled with 17 cm of water (22°C) for 5 min. Total number of floating bouts and total time spent floating was recorded. Higher percent time floating is interpreted as an increased depressive-like response. Immediately after testing mice were euthanized and punch samples of BNST were collected from 2 mm-thick slices, as in Experiment 1.

### Experiment 4: acute effects of antidepressants in males and females following social defeat stress

Male and female mice underwent social stress as described in Experiment 1. Each mouse was randomly assigned to be treated with either a single vehicle injection or sertraline (Figure 1, 5 mg/kg). This dose was based on results from Experiment 3, which showed that 5 mg/kg sertraline significantly increased social interaction. Mice were tested in the social interaction test 30 min following injection, after which they were returned to their home cages for an additional 30 min before the start of forced swim. Mice were euthanized immediately after the forced swim test.

### Experiment 5: effects of the selective TrkB antagonist ANA-12 in control and stressed females

Females were exposed to three episodes of social defeat or control conditions (Figure 1), and were implanted with guide cannulae 1 week later (Plastics One, Roanoke, VA). Guides were aimed at the anterior BNST (anteriorposterior: 0.45 mm; mediolateral: ±1.0 mm; dorsoventral: 5.6 mm) and secured to the skull using surgical screws and acrylic dental cement as previously described (Campi et al., 2014). One week later, half of the mice received either a bilateral infusion of 3 μg of ANA-12 in 200 nL dimethyl sulfoxide (DMSO) or DMSO only. ANA-12 is a small molecule nonpeptide TrkB antagonist (Cazorla et al., 2011), and we used DMSO as a vehicle because it is insoluble in aqueous solutions. We compared social interaction behavior in females naive to defeat that were infused with DMSO (mean ± *SE*, 104.7 ± 8.9, *n* = 10) to those infused with aCSF (102.0 ± 9.7, *n* = 26) and found no significant difference. These data suggest that the use of DMSO vehicle does not interfere with behavioral measurements. Twenty-four hours after infusion, each mouse was tested in social interaction tests. The delay between infusion and testing was based on previous work showing that ANA-12 affects food intake over a 24 h period (Spaeth et al., 2012). One day after the first behavior test, mice received either DMSO or ANA-12 infusion, and they were retested in the social interaction test the following day with a different stimulus female. Needle placement was confirmed with Nissl stained sections (Figures 7C,F).

### Statistical analyses

The distribution of each data set was checked for normality using Q-Q plots and histograms. Except where indicated, behavioral data were analyzed using Two-Way ANOVA testing for effects of stress and sex. Due to heterogeneity of variance, BDNF immunohistochemistry data from Experiment 1 was log transformed. In Experiment 3 social interaction scores were not normally distributed and could not be corrected with log transformations. Thus, these data were analyzed with nonparametric statistics. Data for BDNF expression from Western blots across all three experiments were also analyzed with nonparametric statistics. We used Spearman correlations to examine relationships between BDNF protein expression and behavior by treatment and sex. We corrected for multiple correlations using the Benjamini and Hochberg false discovery rate (FDR) procedure. For Experiment 5, we used paired *t*-tests to compare behavior following infusions of ANA-12 or DMSO.

## Results

### Experiment 1

#### Social defeat has sex-specific effects on behavior and BDNF

**Figure 2:**
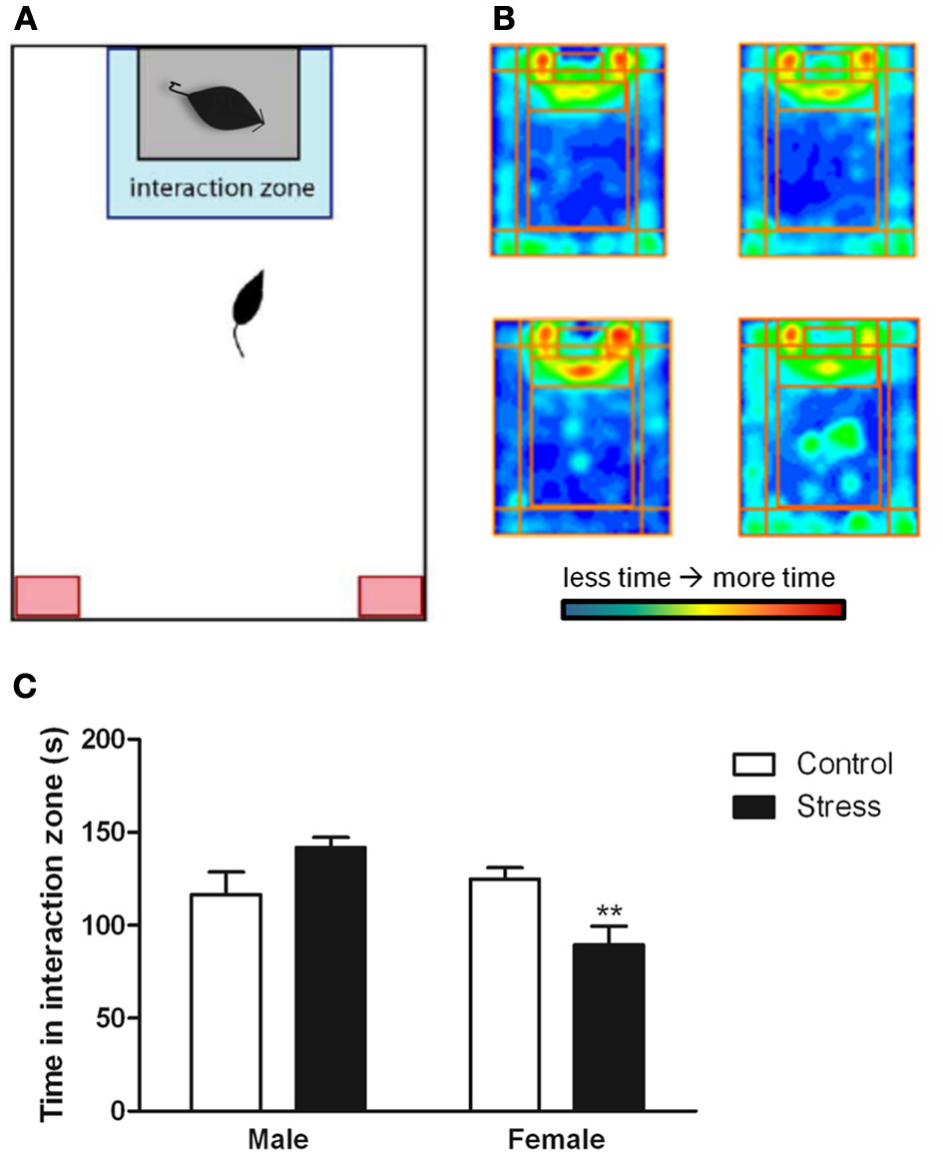
**Exposure to social defeat stress decreased the amount of time female mice but not males spent in the interaction zone interacting with a novel target mouse. (A)** The illustration describes the location of the “interaction zone” (highlighted in blue) and the “corners zone” (highlighted in red) within the social interaction arena. **(B**, clockwise from top left**)** Heat maps depicting space usage during the social interaction phase for control males, control females, stressed females, and stressed males. **(C)** Quantification of time spent in the interaction zone during social interaction phase. Data are shown as mean ± *SE*. (^**^p < 0.01 vs. control), *n* = 13–20 per group.

**Figure 3:**
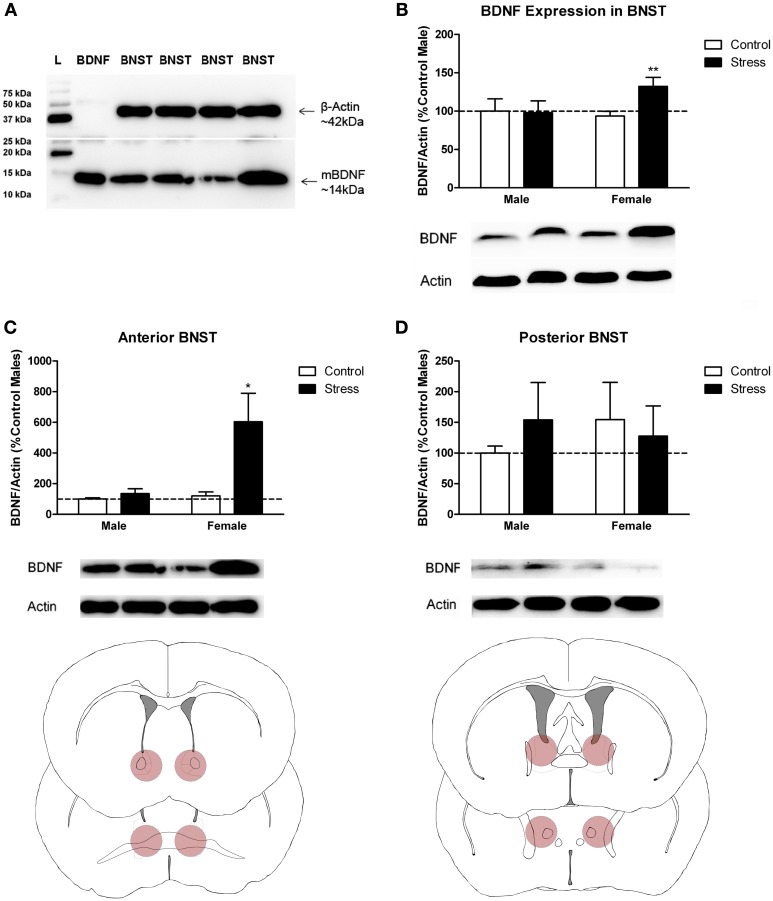
**Social stress increased brain-derived neurotrophic factor (BDNF) protein in females but not males in the bed nucleus of the stria terminalis (BNST). (A)** The anti-BDNF antibody is specific to BDNF protein as evaluated by Western blot using microdissections from California mouse brain bed nucleus of stria terminalis (BNST). The same blot was stained for β-Actin (top half) and BDNF (bottom half). A recombinant BDNF peptide was used as a positive control for the BDNF antibody and negative control for the β-Actin antibody. The Bio-Rad Precision Protein Plus Ladder (L) was run in the first lane. **(B)** BDNF protein in the BNST was significantly increased in stressed females. Western blot analysis of 2 mm-thick punch sample microdissections from BNST showed that females exposed to stress had significantly increased levels of BDNF protein in BNST, *n* = 12–19 per group. **(C,D)** Western blots from 500 micron-thick punches revealed increased BDNF expression specifically in anterior BNST of stressed female mice, *n* = 8–10 per group (representative image for panel C was cropped from full blot presented in panel A). Schematics diagramming tissue punch sites for BNST subdivisions are presented below blots. Illustrations are based on artwork from Paxinos and Franklin (2002), with permission from Academic Press. %BDNF was calculated from the ratio of BDNF:Actin normalized to control mice across blots. Data are shown as mean ± *SE*. (^*^p < 0.05, ^**^p < 0.01 vs. control). Blots have been cropped for clarity to create representative gel pattern and analysis of BDNF levels in BNST.

**Figure 4:**
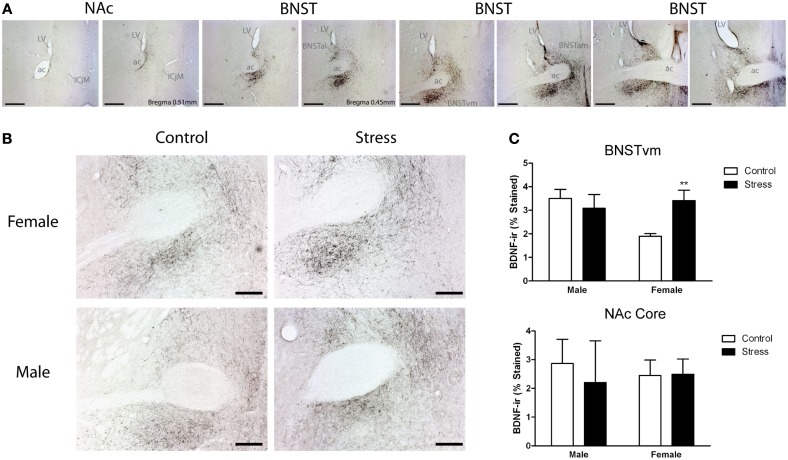
**Social defeat stress increased brain-derived neurotrophic factor (BDNF) immunoreactivity in females but not males in the anterior bed nucleus of the stria terminalis (BNST). (A)** BDNF immunostaining from anterior-posterior serial sections in a representative stressed female, starting in nucleus accumbens (NAc) and continuing through to posterior BNST. Lower power photomicrographs, scale bar = 500 μm. Anterior commissure (ac), lateral ventricle (LV), island of Calleja major (ICjM), and fornix (f) labeled for reference. Bregma coordinates are based on cannula placements in this study and Campi et al. (2014). Puncta were observed in the anterior ventromedial BNST (BNST_VM_), anteriorlateral BNST (BNST_AL_) and anteriormedial BNST (BNST_AM_) **(B)** Representative photomicrographs of BDNF immunostaining within BNST_VM_ from which BDNF immunoreactivity was quantified. Higher power photomicrographs, scale bar = 200 μm. **(C)** Quantification of immunoreactivity (BDNF-ir) revealed increased BDNF in BNST_VM_ (top graph) in stressed female mice but no differences in NAc core (bottom graph). Raw data are shown as %staining. Data are shown as mean ± *SE*. (^**^p < 0.01 vs. control), *n* = 3–5 per group.

**Figure 5:**
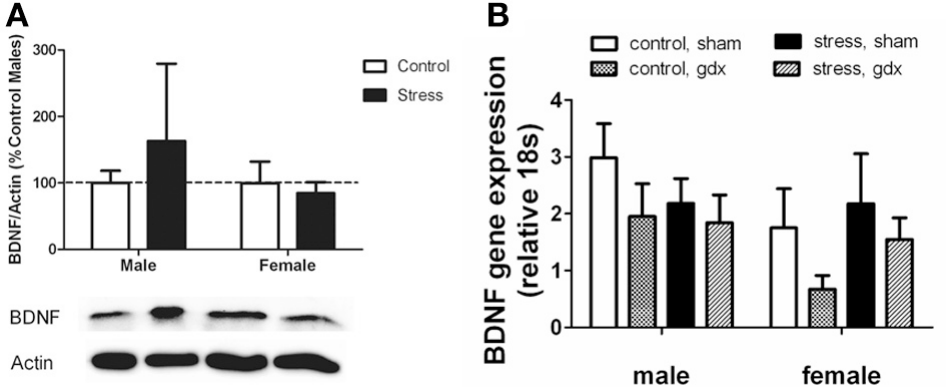
**(A)** Nucleus accumbens (NAc) BDNF protein as evaluated by Western blot analyses. Social defeat had no effect on brain-derived neroutrophic factor (BDNF) in female or male mice. Blots have been cropped for clarity. %BDNF was calculated from the ratio of BDNF:Actin normalized to control mice across blots. Data are shown as mean ± *SE*. *n* = 5–8 per group. **(B)** There were no significant differences in *Bdnf* mRNA as evaluated by real-time PCR analyses using microdissections of bed nucleus of the stria terminalis (BNST) from intact and gonadectomized male and female California mice.

Stressed female mice spent significantly less time than control females in the interaction zone with a novel target mouse, whereas there was no effect of defeat in males [Figure 2, *F*_(1, 62)_ = 11.37, *p* < 0.001]. This effect was specific to social contexts as there were no differences in time spent in the interaction zone during the acclimation or OFT (Table 2). Patterns of BDNF protein expression mirrored social interaction behavior. In punch samples of the entire BNST, stressed females had significantly more BDNF compared to control females [Figure 3B, Mann-Whitney *U* = 76.50, *p* < 0.01]. Overall, BDNF in BNST was negatively correlated with time spent interacting with a target mouse during the social interaction phase (*r* = −0.324, *p* < 0.01). Interestingly, when broken down by groups, stressed females were the only group of mice in which this correlation was significant (*r* = −0.519, FDR adjusted *p* < 0.05). This suggests that individual variation in behavior is linked to individual variation in BDNF expression. Analyses from thinner punch samples showed that stress increased BDNF nearly six-fold in the anterior BNST for females but not males (Figure 3C, Mann-Whitney *U* = 8.00, *p* < 0.05). In posterior BNST, BDNF signals were weaker than anterior BNST, and there were no significant differences (Figure 3D). Immunohistochemistry showed that in the anterior BNST, BDNF-positive puncta and fibers were concentrated in the ventral portions of the region (Figure 4A), including BNST_AM_ and BNST_VM_. Stressed female mice had significantly more BDNF immunoreactivity within the anterior BNST_VM_ compared to control females [Figures 4B,C, *F*_(1, 12)_ = 7.24, *p* < 0.05] whereas there was no effect of stress in males (Figures 4B,C). In the BNST_AM_, stressed females had on average twice as much immunoreactivity than control females, but this difference was not significant [*F*_(1, 12)_ = 2.52, *p* = 0.14, Table 3]. There were no significant differences in BDNF percent-staining in the NAc shell or core, or BNST_AL_ (Figure 4C; Table 3). Consistent with immunostaining results (Figure 4A), BDNF signals were weak within NAc, and defeat had no effect on BDNF protein measured by Western blot (Figure 5A).

**Table 2:** **Effects of stress and sex on behavior during the social interaction test**.

**Social interaction test**	**Male Control**	**Male Stress**	**Female Control**	**Female Stress**
**ACCLIMATION AND INTERACTION**				
Time in interaction zone (empty)	94.0 ± 12.2	95.7 ± 8.1	100.7 ± 9.1	96.4 ± 8.1
Time in interaction zone (target)	116.3 ± 12.2	141.8 ± 19.4	124.8 ± 6.1	89.5 ± 9.9^**^
Time in corners zone (empty)	9.4 ± 4.1	11.7 ± 2.2	9.3 ± 3.1	7.8 ± 2.9
Time in corners zone (target)	8.1 ± 4.7	1.3 ± 1.8	8.4 ± 12.2	8.4 ± 9.5
Total distance (empty)	20.5 ± 3.7	25.1 ± 2.2	25.9 ± 3.2	29.0 ± 6.3
Total distance (target)	19.1 ± 2.6	29.2 ± 5.1	24.3 ± 2.7	30.6 ± 8.9
**OPEN FIELD**				
Time in center zone	32.5 ± 3.4	30.3 ± 2.8	32.7 ± 3.5	28.11 ± 3.0
Time in sides zone	84.5 ± 2.6	86.2 ± 2.8	76.5 ± 3.8	81.5 ± 3.2
Total distance	18.7 ± 3.5	16.3 ± 2.6	20.3 ± 2.9	16.4 ± 3.0

All times in sec, all distances in m.

Data are shown as mean ± SE.

** p < 0.01, treatment difference.

**Table 3:** **Effects of stress and sex on BDNF immunoreactivity**.

**Region**	**Male Control**	**Male Stress**	**Female Control**	**Female Stress**
NAc core	2.9 ± 0.8	2.2 ± 1.5	2.5 ± 0.5	2.5 ± 0.5
NAc shell	1.8 ± 0.6	1.0 ± 0.6	1.3 ± 0.5	0.9 ± 0.4
BNST_AM_	5.2 ± 1.0	4.8 ± 0.6	2.6 ± 0.5	5.6 ± 1.6
BNST_AL_	2.9 ± 0.3	2.1 ± 0.5	1.9 ± 0.5	1.2 ± 0.3
BNST_VM_	3.5 ± 0.4	3.1 ± 0.6	2.0 ± 0.1	3.4 ± 0.4^**^

BNDF-ir quantified as %Staining.

Data are shown as mean ± SE.

** p < 0.01, treatment difference.

### Experiment 2

#### Social defeat did not affect BDNF mRNA in the BNST

Intriguingly, effects of stress were limited to BDNF protein levels, as there were no effects of defeat on *Bdnf* mRNA in the BNST (Figure 5B). We also did not observe any sex differences or effects of gonadectomy on *Bdnf* mRNA. The corresponding behavioral data for these samples are reported in Trainor et al. (2013).

### Experiment 3

#### Sex differences in dose response effects of sertraline on behavior

**Figure 6:**
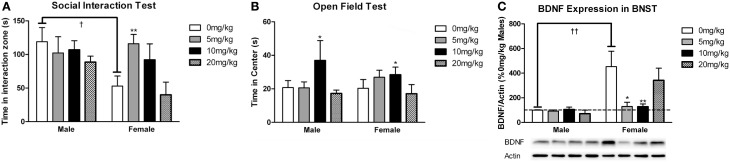
**Measurements of time spent in (A) the interaction zone during the interaction phase of the social interaction test and (B) the center zone of an open field test (OFT) following chronic administration of a selective serotonin reuptake inhibitor (SSRI) antidepressant**. The SSRI sertraline reversed avoidance behavior in stressed female mice when administered at a low dose (5 mg/kg). A moderate dose of sertraline (10 mg/kg) had an anxiolytic effect on time spent in the center of the OFT in male and female mice. **(C)** Low to moderate doses of sertraline reversed increases in brain-derived neurotrophic factor (BDNF) protein in the bed nucleus of the stria terminalis (BNST) in stressed females. 0 mg/kg female mice had significantly more BDNF in BNST than 0 mg/kg male mice, 5 mg/kg female mice and 10 mg/kg/day female mice. Blots have been cropped for clarity. %BDNF was calculated from the ratio of BDNF:Actin normalized to 0 mg/kg mice across blots. Data are shown as mean ± SE (^†^*p* < 0.05, ^††^*p* < 0.01 sex difference, ^*^p < 0.05, ^**^p < 0.01 effects of sertraline), *n* = 5–7 per group.

Sertraline had significant effects on social interaction behavior in females (Figure 6A, Kruskal-Wallis *H*_3_ = 7.56, *p* < 0.05) but not males (*p* > 0.05). Females treated with 5 mg/kg of sertraline spent significantly more time in the interaction zone with a target mouse compared to vehicle treated females (Figure 6A, Mann-Whitney *U* = 3.00, *p* < 0.01). The effect of the 5 mg/kg/day dose was specific to social contexts and was not accompanied by changes in time in the interaction zone during the acclimation phase or in locomotor behavior (Table 4). Consistent with Experiment 1, stressed males treated with vehicle spent significantly more time interacting with a target mouse than stressed females treated with vehicle (Figure 6A, Mann-Whitney *U* = 6.00, *p* < 0.05). During the open field test, there was a significant main effect of sertraline treatment on time spent in the center [*F*_(3, 43)_ = 2.92, *p* < 0.05]. Males and females treated with 10 mg/kg/day spent more time in the center zone compared to 0 mg/kg/day mice (Figure 6B). There were no significant differences in sucrose anhedonia or forced swim test observations (Table 5). However, when averaging sucrose consumption across the first 2 days of testing, there was a nonsignificant trend for increased sucrose consumption in female mice due to sertraline treatment [*F*_(3, 43)_ = 3.012, *p* = 0.05]. A contrast showed that females receiving any dose of sertraline consumed significantly more sucrose than females treated with vehicle (*p* < 0.01). There were also no differences in body weight change following sertraline administration (Table 6).

**Table 4:** **Effects of chronic sertraline and sex on behavior during the social interaction test**.

**Social interaction test**	**Male 0 mg/kg**	**Male 5 mg/kg**	**Male 10 mg/kg**	**Male 20 mg/kg**	**Female 0 mg/kg**	**Female 5 mg/kg**	**Female 10 mg/kg**	**Female 20 mg/kg**
**ACCLIMATION AND INTERACTION**								
Time in interaction zone (empty)	71.0 ± 11.9	85.5 ± 13.1	95.3 ± 9.9	78.5 ± 9.6	50.9 ± 14.1	80.3 ± 16.7	103.9 ± 14.3	74.9 ± 16.9
Time in interaction zone (target)	119.1 ± 21.0	102.3 ± 24.2	106.9 ± 13.6	88.7 ± 8.9	53.0 ± 15.0^†^	116.1 ± 13.7^**^	92.0 ± 23.8	40.0 ± 19.0
Time in corners zone (empty)	12.2 ± 5.3	5.3 ± 2.3	4.1 ± 1.6	16.4 ± 6.9	38.8 ± 18.6	6.6 ± 1.5	5.2 ± 2.0	16.5 ± 10.6
Time in corners zone (target)	18.4 ± 12.8	32.6 ± 27.7	1.6 ± 0.5	13.0 ± 6.8	57.6 ± 31.5	4.1 ± 2.4	12.9 ± 5.5	76.7 ± 32.8
Total distance (empty)	26.5 ± 3.9	17.7 ± 4.6	31.2 ± 8.2	21.6 ± 2.4	29.6 ± 7.1	46.7 ± 6.7	18.1 ± 2.4	19.5 ± 4.9
Total distance (target)	26.2 ± 3.4	19.5 ± 3.3	30.5 ± 5.8	15.9 ± 6.7	41.6 ± 9.0	24.0 ± 6.0	24.0 ± 6.0	30.1 ± 6.9
**OPEN FIELD**								
Time in center zone	20.1 ± 4.1	20.6 ± 3.6	36.9 ± 11.9^*^	17.3 ± 2.0	20.4 ± 5.2	26.9 ± 4.2	28.4 ± 4.6^*^	17.1 ± 5.5
Time in sides zone	111.4 ± 13.0	120.1 ± 18.6	72.8 ± 21.5	119.1 ± 32.5	119.7 ± 12.1	108.1 ± 16.7	111.7 ± 13.8	135.8 ± 17.2
Total distance	17.8 ± 5.6	15.6 ± 4.9	21 ± 7.2	22.1 ± 6.2	21.0 ± 7.7	24.3 ± 5.9	16.5 ± 3.8	12.2 ± 5.3

Data shown as mean ± SE.

Times in sec, distances in m.

^†^p < 0.05, sex difference.

* p < 0.05,

** p < 0.01, trt difference.

**Table 5:** **Effects of chronic sertraline and sex on behavior during tests of antidepressant function**.

**Tests**	**Male 0 mg/kg**	**Male 5 mg/kg**	**Male 10 mg/kg**	**Male 20 mg/kg**	**Female 0 mg/kg**	**Female 5 mg/kg**	**Female 10 mg/kg**	**Female 20 mg/kg**
**SUCROSE ANHEDONIA**								
Day 1 %sucrose consumption	63.6 ± 4.3	63.9 ± 3.3	67.7 ± 9.4	60.5 ± 6.8	50.3 ± 4.9	68.2 ± 4.0	65.9 ± 2.2	65.7 ± 3.1
Day 2 %sucrose consumption	73.0 ± 3.8	66.8 ± 5.1	65.0 ± 4.4	70.7 ± 3.0	63.5 ± 5.6	71.5 ± 4.5	71.1 ± 3.5	69.1 ± 3.4
Average %sucrose consumption	68.3 ± 3.4	65.4 ± 3.2	66.3 ± 5.4	65.6 ± 4.7	56.9 ± 4.4	69.9 ± 3.5	68.5 ± 2.4	67.4 ± 3.0
**FORCED SWIM TEST**								
Immobility time	30.3 ± 20.7	27.2 ± 17.1	15.0 ± 7.9	79.5 ± 71.4	72.0 ± 38.6	37.8 ± 12.6	16.0 ± 8.7	35.6 ± 15.4

Data are shown as mean ± SE.

All times in sec.

% sucrose = sucrose/(sucrose + water) ^*^100.

**Table 6:** **Effects of chronic sertraline on weight gain**.

**Sex, dose**	**Week 0 (g)**	**Week 1 (%)**	**Week 2 (%)**	**Week 3 (%)**	**Week 4 (%)**
Male, 0 mg/kg	36.9 ± 2.6	0.1 ± 1.2	−0.2 ± 1.4	−1.5 ± 2.1	1.1 ± 2.6
Male, 5 mg/kg	37.1 ± 2.9	1.2 ± 1.3	0.0 ± 0.3	0.1 ± 1.2	1.6 ± 1.1
Male, 10 mg/kg	38.1 ± 3.5	−1.7 ± 2.7	−3.2 ± 1.4	0.8 ± 2.5	0.8 ± 2.3
Male, 20 mg/kg	38.8 ± 3.3	0.8 ± 1.7	−1.6 ± 0.6	0.9 ± 1.3	0.7 ± 1.5
Female, 0 mg/kg	38.7 ± 3.6	−1.0 ± 1.3	−1.2 ± 0.5	−1.8 ± 1.1	1.6 ± 0.8
Female, 5 mg/kg	38.9 ± 2.3	−1.9 ± 1.2	0.9 ± 2.1	2.1 ± 2.0	2.3 ± 2.7
Female, 10 g/kg	35.7 ± 2.0	0.6 ± 0.8	2.8 ± 1.6	1.6 ± 1.7	2.1 ± 2.0
Female, 20 mg/kg	37.3 ± 1.9	0.1 ± 2.0	−0.6 ± 2.5	0.9 ± 2.3	2.9 ± 3.0

Data are shown as mean ± SE.

#### Sex differences in effects of sertraline on BDNF in BNST

Sertraline had a significant effect on BDNF expression in BNST of females (Figure 6C, Kruskal-Wallis *H*_3_ = 9.847, *p* < 0.05) but not males (*p* > 0.05). In females the 5 mg/kg (Mann-Whitney *U* = 3.00, *p* < 0.05) and 10 mg/kg (Mann-Whitney *U* = 2.00, *p* < 0.01) doses reduced BNST BDNF expression compared to vehicle. There was a significant negative correlation between BNST BDNF and interaction times with a target mouse during social interaction (*r* = −0.390, *p* < 0.01). Females treated with vehicle had significantly more BDNF protein in BNST than males treated with vehicle (Figure 6C, Mann-Whitney *U* = 0.00, *p* < 0.01).

### Experiment 4

#### Acute effects of sertraline on behavior and BDNF

There was no effect of sex or a single treatment of sertraline on time spent in the interaction zone with a target mouse. Neither sex nor sertraline had an effect on any other variables during the acclimation or interaction phases (Table 7). Additionally, neither sex nor dose had an effect on time spent in the center or sides zones of the OFT. However, male mice did travel a significantly greater distance during OFT than female mice [*F*_(1, 13)_ = 6.77, *p* < 0.05]. There was a significant effect of sertraline on immobility time during the forced swim test [*F*_(1, 13)_ = 2.92, *p* < 0.05], with sertraline increasing floating behavior in both males and females (Table 7, *p* < 0.05). Acute sertraline administration had no significant effect on BDNF expression in BNST.

**Table 7:** **Effects of acute sertraline on male and female behavior**.

**Social interaction test**	**Male 0 mg/kg**	**Male 5 mg/kg**	**Female 0 mg/kg**	**Female 5 mg/kg**
**ACCLIMATION AND INTERACTION**				
Time in interaction zone (empty)	78.1 ± 15.5	84.6 ± 9.8	76.2 ± 9.4	55.4 ± 14.4
Time in interaction zone (target)	54.0 ± 22.5	44.7 ± 20.7	50.2 ± 23.8	55.0 ± 19.6
Time in corners zone (empty)	5.3 ± 2.0	6.0 ± 1.9	10.1 ± 2.3	12.2 ± 5.4
Time in corners zone (target)	3.7 ± 1.7	4.3 ± 1.3	24.1 ± 10.2	37.8 ± 28.3
Total distance (empty)	22.4 ± 6.3	17.8 ± 2.7	18.6 ± 1.6	25.3 ± 5.2
Total distance (target)	18.0 ± 6.5	13.0 ± 3.2	20.1 ± 6.1	19.7 ± 5.1
**OPEN FIELD**				
Time in center zone	40.3 ± 7.3	44.3 ± 7.7	28.8 ± 4.2	46.2 ± 12.7
Time in sides zone	66.4 ± 9.8	72.1 ± 9.4	92.4 ± 12.0	73.1 ± 11.2
Total distance	28.8 ± 2.3^†^	25.2 ± 1.3	20.3 ± 2.2	20.3 ± 3.1
**FORCED SWIMMING**				
Immobility time	14.5 ± 5.0	53.5 ± 17.5^*^	10.3 ± 2.8	24.7 ± 11.5^*^

Data are shown as mean ± SE.

All times in sec, all distances in m.

† p < 0.05, sex difference.

* p < 0.05, dose effect.

n = 4–6 per group.

### Experiment 5

#### Effects of ANA-12 infusion on social interaction

**Figure 7:**
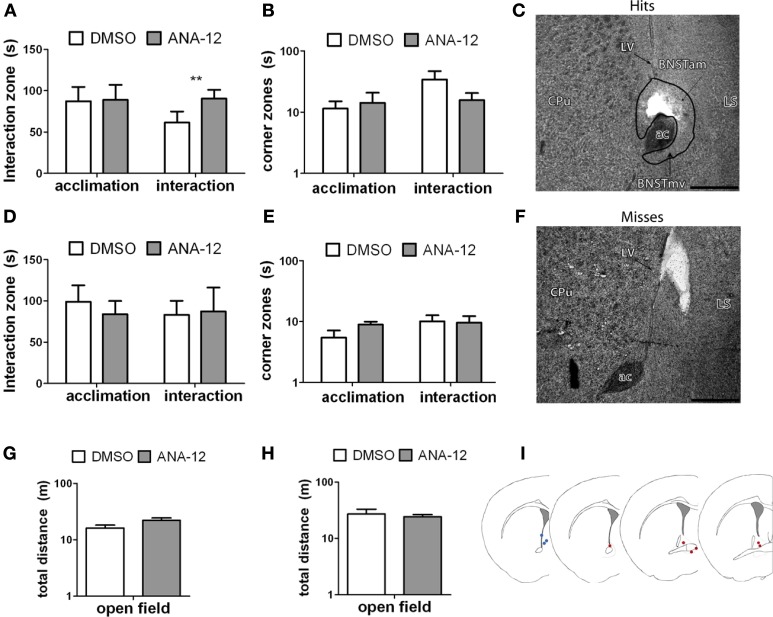
**(A,B,G)** Effects of ANA-12 on behavior for stressed female mice in which needle tracks hit the anterior bed nucleus of the stria terminalis (BNST). ANA-12 increased time spent interacting with a novel mouse (**A**; interaction, ^**^paired *t*-test *p* = 0.01) and decreased time spent in the corner zones opposite a novel mouse (**B**; interaction, nonsignificant). There was no effect of ANA-12 on behavior in the absence of a social stimulus (**A**,**B**; acclimation) or on behavior in an open field test **(G)**. **(C)** Representative image of needle track hitting the anterior bed nucleus of the stria terminalis. Structures caudate-putamen (CPu), anterior commissure (ac), anterior-medial bed nucleus of the stria terminalis (BNST_am_), ventromedial bed nucleus of the stria terminalis (BNST_mv_) and lateral septum (LS) shown for reference. Photos captured at lower power, scale bar = 500 μm. **(F)** Representative image of needle track missing anerior BNST. **(D,E,H)** Effects of ANA-12 on behavior for stressed female mice in which needle tracks missed the anterior bed nucleus of the stria terminalis (BNST). ANA-12 did not affect time spent interacting with a novel mouse (**D**; interaction) or empty cage (**D**; acclimation). There was no effect of ANA-12 on time spent in the corner zones opposite a novel mouse or empty cage **(E)**. There was no effect of ANA-12 on locomotor behavior during an open field test **(H)**. Data are shown as mean ± *SE*, *n* = 4–6 per group. **(I)** Schematic representations of recorded hits (red dots) and misses (blue) from nissl-stained sections of stressed female mice with cannula guides implanted (based on artwork from Paxinos and Franklin (2002), with permission from Academic Press).

For stressed female mice in which needle tracks hit the anterior BNST, ANA-12 infusions significantly increased time spent in the interaction zone in the presence of a novel female [Figures 7A,C, paired *t*_(5)_ = −4.41, *p* < 0.01]. There was also a nonsignificant trend for reduced time in the corners zones following ANA-12 infusions [Figure 7B, paired *t*_(5)_ = 1.643, *p* = 0.16]. There was no effect of ANA-12 on these variables during the acclimation phase (in the absence of a social stimulus, Figure 7A). There was also no effect of ANA-12 on locomotor behavior (Figure 7G) or time spent in the center of the arena during the OFT. For mice in which needle tracks missed the anterior BNST, there were no differences in behavior (Figures 7D–F,H, all *p*'s > 0.5). Three mice received ANA-12 infusions in which cannulas missed the anterior BNST. In these mice time spent in the interaction zone in the presence of a novel female was relatively low after both DMSO (mean ± *SE*, 83.03 ± 17.00) and ANA-12 [87.3 ± 28.78, paired *t*_(2)_ = −0.21, *p* > 0.05] treatment. There was also no difference in time spent in the corners zone [paired *t*_(2)_ = 0.33, *p* > 0.05]. For mice with misplaced needle tracks, the power to detect a difference of 35 sec in the interaction zone (during the social interaction phase) was 82%. There were no differences in behavior following ANA-12 infusions in females naïve to defeat, regardless of needle track placement (Table 8).

**Table 8:** **Effects of ANA-12 in female mice naïve to defeat on behavior during the social interaction test**.

**Social interaction test**	**Hits DMSO**	**Hits ANA-12**	**Misses DMSO**	**Misses ANA-12**
**ACCLIMATION AND INTERACTION**				
Time in interaction zone (empty)	96.8 ± 15.1	97.0 ± 20.9	97.8 ± 21.2	104.2 ± 22.7
Time in interaction zone (target)	91.7 ± 12.0	85.9 ± 17.8	117.8 ± 12.5	95.1 ± 11.4
Time in corners zone (empty)	7.3 ± 2.7	10.5 ± 5.1	9.6 ± 4.5	10.3 ± 5.4
Time in corners zone (target)	8.7 ± 3.0	14.1 ± 5.0	4.4 ± 1.5	9.8 ± 3.9
**OPEN FIELD**				
Time in center zone	28.9 ± 2.5	32.3 ± 11.0	35.5 ± 11.7	23.4 ± 6.3
Total distance	23.3 ± 0.9	27.3 ± 3.0	23.8 ± 5.8	25.1 ± 2.9

All times in sec, all distances in m.

Data are shown as mean ± SE.

n = 5 per group.

## Discussion

We demonstrate that three episodes of social defeat stress increase mature BDNF protein in females within the BNST. The effect of social stress on BDNF protein was robust and long lasting and was specific to the anterior BNST, which is not sexually dimorphic in size in California mice (Campi et al., 2013). Interestingly, increased BDNF protein was not accompanied by changes in *Bdnf* mRNA in the BNST. Chronic, low doses of sertraline that reversed social withdrawal in females also reduced stress-induced increases in BDNF expression. Acute SSRI treatment was not effective, consistent with delayed therapeutic responses to SSRIs in humans (Nierenberg, 2001; Jiao et al., 2011). Finally, infusions of the selective TrkB antagonist ANA-12 into the anterior BNST increased social interaction behavior in stressed females. No effects of ANA-12 infusions were observed in females naïve to defeat, indicating that the effects of BDNF signaling via TrkB are stress-induced. These data suggest that a stress-induced activation of TrkB by BDNF in the anterior BNST is an important mechanism contributing to social withdrawal in female California mice.

### BDNF protein is upregulated in the anterior BNST in female california mice after social defeat

The effect of defeat stress on BDNF was confined to the anterior portion of the BNST. In hamsters, social defeat increases *c-fos* gene expression in anterior subregions of the BNST (Kollack-Walker et al., 1997). Within the anterior BNST, fear conditioning studies have highlighted the importance of the BNST_AL_ (Gray et al., 1993; Walker et al., 2003). For example, chronic mild stress increased *Bdnf* mRNA in the dorsal BNST_AL_ in male rats (Hammack et al., 2009a,b). Interestingly, in Fos-tau-LacZ (FTL) transgenic mice, the BNST_AM_ was the only part of the BNST activated following recall of fear contexts (Ali et al., 2012). Our immunostaining analyses indicated that there was a high concentration of BDNF puncta and fibers in the BNST_AL_ and the BNST_AM_. The BNST_AM_ contains corticotropin-releasing hormone (CRH)-positive neurons (Moga et al., 1989; Phelix and Paull, 1990; Dong and Swanson, 2006) and fibers (Dong and Swanson, 2006), and CRH acting in the BNST increases anxiety-like behavior (Lee and Davis, 1997). We recently demonstrated that social withdrawal responses in California mice can be uncoupled from hypothalamic-pituitary-adrenal (HPA) activity (Trainor et al., 2013). If stress induced changes in BDNF affected CRH expression in PVN, we predict that any behavioral consequences would be independent of HPA activity.

Consistent with immunoblot data, stressed females had more BDNF immunoreactivity than control females in the BNST_VM_. We found stressed females to have greater immunoreactivity than control females in a sampling of the anterior BNST_VM_, and there was a trend for increased immunoreactivity in a sampling of BNST_AM_. These areas of the anterior BNST receive dense projections from the central nucleus of the amygdala (Dong et al., 2001). In the anterior BNST, male mice had more BDNF immunoreactivity than expected based on immunoblotting experiments. Immunohistochemistry analyses are incapable of differentiating between mature and immature forms of BDNF, suggesting that males may have more immature BDNF protein than females. Observations of BDNF immunoreactivity in NAc were consistent with immunoblot results, indicating that stress-induced changes in BDNF do not extend to NAc.

Defeat increased BDNF protein expression in the BNST but had no effect on *Bdnf* mRNA as measured by qPCR. This pattern could reflect post-transcriptional regulation of *Bdnf* mRNA, which has been identified as a key factor contributing to the antidepressant effects of ketamine (Autry et al., 2011). Low doses of ketamine rapidly increase the amount of BDNF protein in hippocampus without affecting *Bdnf* mRNA levels. Although we do not know whether defeat stress rapidly increases BDNF protein in the BNST, our results indicate that this change persists up to 4 weeks. An alternate explanation for this effect is that increased BDNF protein in the BNST reflects changes in axon terminals originating outside the BNST. In Syrian hamsters social defeat increases *Bdnf* mRNA in the basolateral amygdala (BLA) (Taylor et al., 2011), and increased *Bdnf* transcription is observed in the BLA following fear conditioning (Rattiner et al., 2005). Interestingly, the BLA sends excitatory projections to the BNST (Corominas et al., 2010), which may be a means for BDNF release into the BNST.

### TrkB signaling mediates social aversion in female california mice

We found that blocking BDNF action in the anterior BNST with the selective TrkB antagonist ANA-12 also blocked the expression of social aversion in stressed females. This effect occurred when ANA-12 was infused directly into the anterior BNST 24 h prior to social interaction testing (Figures 7A,C). Stressed females receiving off-site infusions of ANA-12 did not display similar increases in social interaction (Figures 7D,F). Given that the anterior BNST is located in proximity to the lateral ventricle, there is a possibility that infusions could enter the ventricle and act elsewhere in the brain. However, histological analysis of a small number of cannula misses showed that some misses were in even closer proximity to the lateral ventricle than hits (Figures 7F,I), yet there was no evidence for any effect of ANA-12 on behavior in animals with misplaced cannulae. Additionally, ANA-12 infusions in females naïve to defeat produced no detectable effects on locomotor behavior, anxiety-like behavior or social interaction behavior (Table 8), suggesting a stress-induced mechanism. It is usually assumed that in the adult brain that BDNF is released by axon terminals and activates TrkB in the post-synaptic cell. However, TrkB has been observed in both dendrites and axon terminals (Drake et al., 1999), raising the possibility that dendritic release of BDNF could alter activity of presynaptic neurons (Helgager et al., 2013).

### Chronic administration of a low dose of SSRI reverses social aversion in females

The low dose of sertraline significantly reduced BDNF expression and increased social interaction in stressed female mice. Both the anterior and the posterior subregions of BNST are innervated by serotonergic projections from the caudal dorsal raphe (Vertes, 1991). Chronic stress alters the serotonin receptor profile in BNST_AL_, leading to increased expression of excitatory serotonin receptor subtypes (Hazra et al., 2012). In this context, our results suggest that further study of the effects of chronic SSRI administration on serotonin receptor expression in the BNST (particularly anterior subregions) could provide important insights into sex differences in the effects of stress on behavior. Although low doses of sertraline reduced BDNF expression and increased social interaction in females, the effects of sertraline on BDNF and social aversion waned at higher doses. Interestingly, clinical studies found that higher doses of SSRIs disrupt activity rhythms of fetuses during pregnancy, but lower doses did not produce these effects (Mulder et al., 2011). High-dose effects of sertraline were not observed in male California mice, which did not display a social withdrawal phenotype following defeat stress. These findings are consistent with previous studies showing that sertraline has a 30% shorter half-life in men vs. women (Ronfeld et al., 1997), and that the magnitude of behavioral response to SSRIs is twice as strong in women compared to men (Khan et al., 2005). These results demonstrate the potential importance of sex differences when determining dosage regiments of SSRIs.

We did not test the effects of sertraline on mice naïve to defeat, which raises the possibility that the observed effects of sertraline would have occurred in the absence of defeat stress. Previous work and our own results suggest this is very unlikely. First, a robust finding from studies of SSRIs is that these pharmaceuticals have no effect on social interaction behavior (Cao et al., 2010; Vialou et al., 2010; Warren et al., 2012) or sucrose consumption (Papp et al., 2002) in animals that have not been exposed to stress. Second, our results show that females treated with the low dose of the sertraline (5 mg/kg) demonstrated social interaction levels (~100 s) that were indistinguishable from a population mean of over 100 control females tested in the social interaction test (Trainor et al., 2013). Finally, ANA-12 infusions had no effect on behavior in females naïve to defeat, consistent with previous studies documenting a lack of antidepressant efficacy in animals that lack a stress-induced behavioral phenotype.

We observed weaker effects of sertraline on sucrose intake and forced swim. On average, females treated with sertraline consumed more sucrose than vehicle treated animals. However, sertraline did not reduce floating behavior in the forced swim test as predicted. Sucrose anhedonia and forced swim are generally accepted as valid paradigms for measuring antidepressant action, although results from these tests can sometimes be inconsistent (Cryan and Mombereau, 2004; Treadway and Zald, 2011). Strain differences are the most common explanation for variable effects of SSRIs on forced swimming in mice (Carr and Lucki, 2011). For example, male and female BALB/CJ mice displayed less immobility following chronic administration of the SSRI citalopram, while a recent study using C57 mice did not (Jiao et al., 2011). In California mice the effects of defeat and sertraline appear to be more robust in social contexts.

## Conclusions

In summary, social defeat increases BDNF protein in the anterior BNST of females but not males, and blocking BDNF action via the TrkB receptor blocks stress-induced social withdrawal in females. These results suggest that the understudied anterior subdivisions of BNST could be important nodes mediating sex differences in behavioral responses to stress. Future study is necessary to determine the mechanisms mediating increased BDNF protein, including whether post-transcriptional mechanisms are involved. These results illustrate the importance of examining the effects of stress in both males and females, and considering the diverse effects that neurotrophins have on stress-related behaviors.

### Conflict of interest statement

The authors declare that the research was conducted in the absence of any commercial or financial relationships that could be construed as a potential conflict of interest.
